# Fast Detection of Tomato Sucker Using Semantic Segmentation Neural Networks Based on RGB-D Images

**DOI:** 10.3390/s22145140

**Published:** 2022-07-08

**Authors:** Truong Thi Huong Giang, Tran Quoc Khai, Dae-Young Im, Young-Jae Ryoo

**Affiliations:** 1 Department of Electrical Engineering, Mokpo National University, Muan 58554, Korea; tthgiang@ttn.edu.vn (T.T.H.G.); tqkhair@gmail.com (T.Q.K.); 2 Components & Materials R&D Group, Korea Institute of Industrial Technology, Gwangju 61012, Korea; dylim@kitech.re.kr; 3 Department of Electrical and Control Engineering, Mokpo National University, Muan 58554, Korea

**Keywords:** tomato sucker detection, tomato pruning, semantic segmentation neural network, real-time, RGB-D

## Abstract

Tomato sucker or axillary shoots should be removed to increase the yield and reduce the disease on tomato plants. It is an essential step in the tomato plant care process. It is usually performed manually by farmers. An automated approach can save a lot of time and labor. In the literature review, we see that semantic segmentation is a process of recognizing or classifying each pixel in an image, and it can help machines recognize and localize tomato suckers. This paper proposes a semantic segmentation neural network that can detect tomato suckers quickly by the tomato plant images. We choose RGB-D images which capture not only the visual of objects but also the distance information from objects to the camera. We make a tomato RGB-D image dataset for training and evaluating the proposed neural network. The proposed semantic segmentation neural network can run in real-time at 138.2 frames per second. Its number of parameters is 680, 760, much smaller than other semantic segmentation neural networks. It can correctly detect suckers at 80.2%. It requires low system resources and is suitable for the tomato dataset. We compare it to other popular non-real-time and real-time networks on the accuracy, time of execution, and sucker detection to prove its better performance.

## 1. Introduction

Many smart technologies have been applied in agriculture [1]. “Smart farm” has become a common word which refers to farms applying intelligent and autonomous technologies such as IoT (Internet of Things) and AI (Artificial Intelligence) on crop monitoring and robots for post-harvesting. Tomato is an important horticultural crop and is popular in smart farms in many countries. Many types of research related to tomatoes are made to increase the tomato quality and production, such as leaf disease detection [2,3,4,5]. These researchers used different deep convolution neural networks to classify the tomato leaf images to different types of diseases. There is also other research such as tomato fruit detection and counting [6], tomato flower detection and counting [7], tomato leaf area estimation [8], and tomato harvesting by a robot [9].

**Figure 1:**
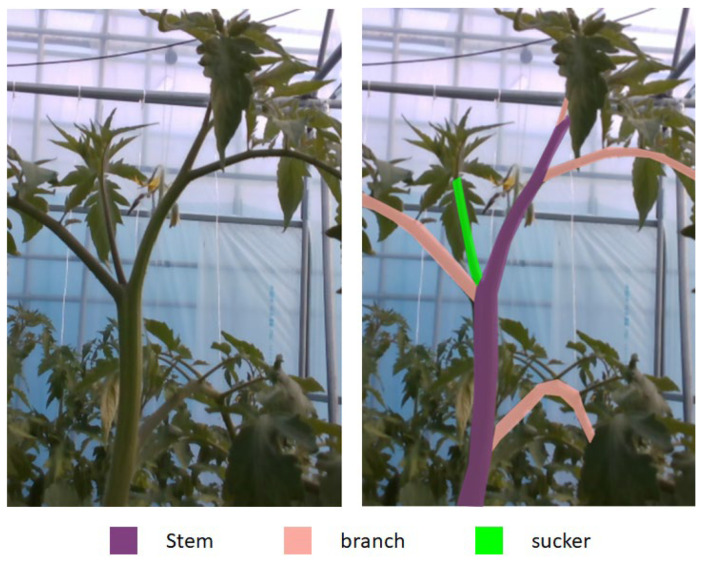
Tomato plant parts. A stem is a part that has nodes from that suckers, leaves, and flowers grow out. The first stem forms the main axis of the plant. A tomato plant should have one to three stems. We call a compound leaf which only has small leaves a branch. A sucker rises at a node of a stem. It is always between a stem and a branch. If the sucker is not removed and is old enough, it becomes a stem.

Tomato sucker pruning is an important activity, but there is no public research about how to remove tomato suckers automatically. Figure 1 shows an example of a tomato sucker which is green in the right image of Figure 1. A sucker is always between a stem and a branch. It should be cut off when it is small before it becomes a stem. Usually, farmers remove suckers from their experiences. In 2007, Ara et al.’s research [10] demonstrated that pruning affects not only yield but also fruit characters. Their experiments showed that tomato yield is much higher, tomato fruit width is larger, and tomato fruit wall is thicker with pruning. Pruning also has a positive affection on the disease and insect infestation of tomato plants [11]. Because of these benefits, we study this subject.

The research on grape pruning [12] or apple pruning [13] showed that branches should be segmented before finding a pruning point. Therefore, in this research, we studied an approach for sucker tomato detection by semantic segmentation neural network. The result of this neural network shows which parts of images are suckers. With this result, other algorithms can be applied to find the position of sucker cut-off points in the future. This is the primary step for future tomato pruning by robots automatically.

Before building our own semantic segmentation neural network, we surveyed current semantic segmentation neural networks such as UNet [14], SegNet [15], and Deeplab V3 [16]. Although these networks can reach high accuracy, their numbers of parameters are high, which leads to intensive device memory. We will implement this AI program on embedded or low-resource systems so that this is a weak point of these networks. On the other hand, some real-time image semantic segmentation networks research has been public recently, such as ICNet [17], BiSeNet [18], and Fast-SCNN [19]. However, these networks do not meet the accuracy requirements. We get pressure on both accuracy and time of execution. This paper proposes a new semantic segmentation neural network that can run in real-time on low-memory GPU with high performance. We used Depth-wise Convolution [20] to reduce computation. We also used a Bottleneck residual block [21], which is used commonly in real-time networks, to extract features of images. To get the global context feature, which is quite important to distinguish tomato plant parts, a Pyramid Pooling block [22] was added to the network. Moreover, we adopted a deep layer cascade (LC) method [23] to optimize the network’s output. We find that decreasing the resolution of layers leads to losing a lot of information with our dataset. Furthermore, we cannot increase the feature channels too much because it costs memory and time. Therefore, we focused on different ways to extract features. We implemented three different feature blocks to get image features and keep the number of feature channels low.

**Figure 2:**
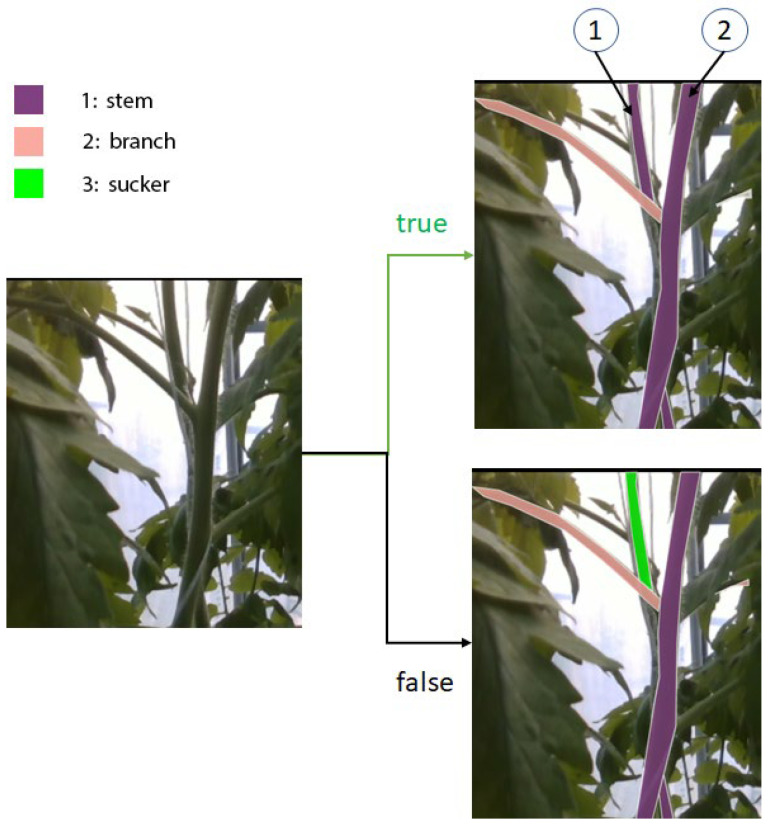
An example of a misunderstanding. The stem (1) can be recognized as a sucker because its position is between a branch and the stem (2) in the RGB image.

For training, evaluating, and comparing the proposed neural network with other networks, we made an RGB-D tomato dataset. Although RGB-D images gain a lot of attention in the recognition task of computer vision, they are mostly applied to indoor semantic segmentation research [24,25,26] or salient object detection research [27,28,29]. The interesting part of the research is how to fuse the feature of depth images and RGB images. Although the moment and the way fusion happens may be different, the processes that extract RGB and Depth image features are usually separate. Our research works on tomato plant parts which are very closed to each other. Leaves take an especially high appearance rate and cover the other tomato parts. The depth information is not that different between adjacent objects. We did some experiments using complete depth information and RGB images as inputs. Their results are not accurate. However, the depth information can help us to separate the close and the far tomato plants when they are captured in one image. Sometimes, in an RGB image, a stem crosses the other stem at its node like in the left image of Figure 2. In reality, stem 2 is further than stem 1 if we look from the camera. From the viewpoint of the 2D image, stem 1 is between a branch and stem 2. It is similar to a sucker and is often recognized as a sucker by many models. We want to use depth information to reduce this misunderstanding. The depth or the distance of a sucker to the camera is often similar to that of its stem. In the example of Figure 2, the depth of stem 1 is higher than the depth of stem 2. This information gives the network more data to recognize that stem 1 is not a sucker, and it helps the model distinguish a real sucker from other parts. Therefore, we propose a new method for fusion depth information. We do not use full-depth information. We only use the depth information for objects that models may have difficulty predicting.

Finally, our research works on RGB-D images, so we designed a suitable structure for depth image processing to efficiently exploit the depth information. Our model has a better balance between accuracy and time of execution.

In summary, our contributions are:

1. We propose a semantic segmentation neural network. This real-time network can be executed on low GPU devices with a low number of parameters and has a better balance between accuracy and time of execution to other networks.
2. We propose a two-stage neural network by adapting the idea deep layer cascade (LC) method [23] in a different way which is easier in implementation, training, and applying depth information. It gives remarkable progress in prediction.
3. We propose a method that uses both depth information and RGB images in prediction for better results.
4. We make a new RGB-D dataset with more than 600 images of tomato plants.

## 2. RGB-D Dataset of Tomato Sucker

### 2.1. Setup for RGB-D Image

We used Intel Realsense D435 made in Thailand to get RGB-D images at a resolution of 480 × 640 for both RGB and depth images. We used “aligned_depth_to_color” property of Realsense SDK 2.0 when taking depth images to align RGB and depth images. The camera was handheld and connected to a laptop to generate and store images. We moved the camera around the tomato plants and along the tomato beds to get images. We only selected images containing tomato parts to make the dataset. The average distance between the camera and tomato plants was 0.5 m. We took images from a smart farm over two months at different moments of the day. This made our dataset more general, and our model could be more accurate.

### 2.2. RGB-D Images of Tomato Sucker

Our research target is tomato sucker detection, so each image in our dataset should include suckers, stems, and branches. Some images did not have suckers. An RGB-D image includes a pair of images. One image is a normal RGB image. The other image describing the distance from objects to the camera is called a depth image. It could be a color-visualized image or a greyscale image. In this research, we propose a method to encode depth information to RGB images. Then, the depth information can be easily restored from these encoded images. Each pixel in the RGB image has a distance to the camera position denoted by *d_ij_*. The corresponding pixel in the depth encoded image has the value *R_i,j_*, *G_i,j_*, *B_i,j_* as the red, green, and blue channel.

$$d_{ij}=R_{ij}+G_{ij}\times 256+B_{ij}\times 256\times 256$$

### 2.3. Semantic Segmentation

**Figure 3:**
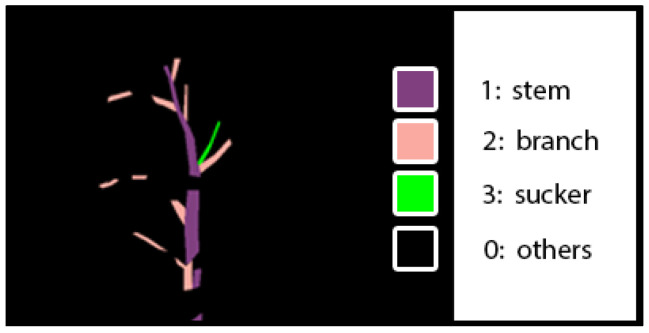
A visualized example of a ground-truth label image.

In our research, we need to know that each pixel in the image belongs to suckers, stems, branches, or others. Therefore, the images were labeled as four classes: stem, branch, sucker, and others, with values 1, 2, 3, and 0, respectively. We focused on how to detect suckers, branches, and stems because we can find the pruning point of the suckers from this information. We did not label the other parts of tomato plants, such as leaves and fruits. We did not detect them in our network. Figure 3 shows a visualized example of a ground-truth label image. Violet regions, pink regions, green regions, and black regions represent stems, branches, suckers, and other objects, respectively.

### 2.4. Dataset

**Figure 4:**
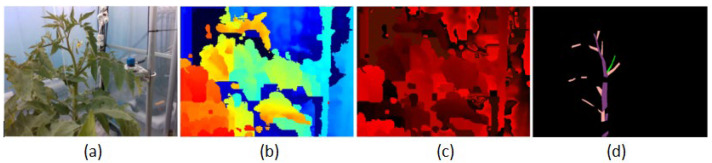
Four types of images in our dataset. (**a**) is RGB image, (**b**) is colored depth image, (**c**) is encoded depth image, and (**d**) is labeled image.

The dataset includes four types of images: RGB images, Depth colorized images, Depth encoded images, and ground truth labels with resolution 480 × 640, as in Figure 4. 

This dataset contains more than 600 images of each type, and we keep updating it date by date. Our images are saved in the PNG extension. We want to make our images in the dataset similar to those taken from the camera. The dataset is easy to verify and explore by humans. This is very helpful for us in training and deploying the application into practice. The labeled images can be seen as black images because they have only one channel and values 0, 1, 2, and 3 corresponding to others, stems, branches, and suckers. To display them as in Figure 4, we need to read a program to map them to RGB images.

## 3. Related Work

### 3.1. The Development of Semantic Segmentation

In the last ten years, Deep Convolutional Neural Networks (DCNNs) outperformed in the task of visual recognition. Especially, in 2012, Alexnet [30] won the ILSVRC-2012 competition, which made a breakthrough in the development of DCNNs. Subsequently, VGGNet [31], GoogLeNet [32], and ResNet [33] continued having big success in image classification tasks and became the backbone network of many other neural networks. Deeplab implemented ResNet as a part of their structure, which was a remarkable semantic segmentation network when it got advanced results on three datasets: PASCAL-Context, PASCAL-Person-Part, and Cityscapes [16]. The most important key points in the Deeplab series use dilated convolution and Atrous Spatial Pyramid Pooling (ASPP), which can capture the features of not only the objects but also the object context at multiple scales. These networks have good results on many benchmark datasets but do not fit with real-time applications and low-memory GPU hardware.

In these years, with the development of robotics and other embedded systems, there have been some well-known real-time networks such as ICNet [17], BiSeNet [18], ContextNet [34], and Fast-SCNN [19]. ICNet uses multiple models with different input resolution images and shares the weight through each model. BisetNet, ContextNet, and Fast-SCNN using Depth-wise Convolution and Inverted Bottleneck Residual blocks which are proposed in MobileNets and MobileNet-V2. Furthermore, the three networks also apply the Pyramid Pooling blocks to capture the global context of the image. These techniques are the key techniques to get real-time performance, but the order and the number of these blocks are different for each model. It depends on the requirement of speed and accuracy. We use these blocks in the second stage of our proposed neural network.

### 3.2. Deep Layer Cascade (LC) Method

**Figure 5:**
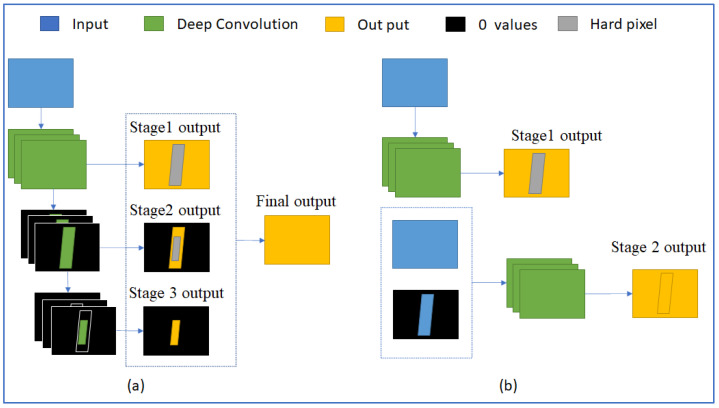
(**a**) is the deep LC structure, and (**b**) is our proposed multi-stage structure. The yellow region is the final output. The grey region is a hard region that is detected by comparing the pixel score of output to a threshold. The black region is the removed region of pixels having 0 value.

The prediction of semantic segmentation neural networks often has some noises or uncertain pixels. These noises always come from similar or complex regions. Xiaoxiao Li et al. proposed a novel deep layer cascade (LC) method to solve this problem [23]. According to the LC method, the network includes three stages. After images go through the first stage, only pixels having a low score in prediction may go to the second stage. The output of the second stage was verified again, only pixels having low scores go through the third stage. The outputs of stage 1, stage 2, and stage 3 only contain high-score pixels. The final output is the combination of the three outputs. Because of Stage 2 and stage 3, the network accuracy is improved. The image Figure 5a in Figure 5 shows the structure of the deep LC method. This method can be applied in different semantic segmentation neural networks.

The output of each stage is a float value matrix corresponding to image pixels. Its values are between 0 and 1. The higher a value is, the more correct this pixel is. Results of low-score pixels are less reliable than results of high-score pixels. The less reliable region is detected by comparing this value to a threshold. Pixels are hard pixels if their values are smaller than this threshold, and pixels are easy pixels if their values are greater than this threshold. The idea of this method is to look more carefully at the hard pixel by passing hard pixels to stage 2 and stage 3.

This is an interesting idea. However, using three stages makes the network heavier. In addition, the time to calculate new input for stage 2 and stage 3 is considerable. Therefore, it is not suitable for real-time purposes.

### 3.3. Fast-SCNN—A Real-Time Semantic Segmentation Neural Network

**Figure 6:**
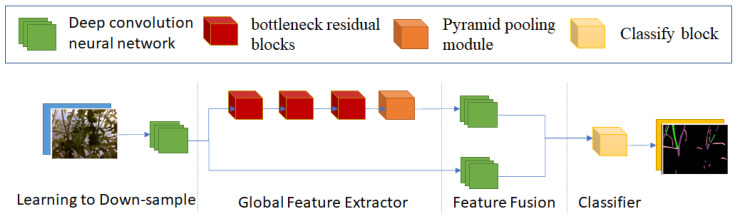
Fast-SCNN structure.

Fast-SCNN is the latest real-time semantic segmentation neural network with high performance on the Cityscape dataset [19]. Its structure is inspired by MobileNets [20] and MobileNets V2 [21] convolution neural networks. Figure 6 shows the structure of Fast-SCNN, including four parts. “Learning to Down-sample” reduces the image resolution. “Global Feature Extractor” is the main part that extracts image features. It uses three different Bottleneck Residual blocks and a Pyramid Pooling block. “Feature Fusion” combines the output of the second and first part before passing through “Classifier”, the final part. Instead of using normal convolution, Fast-SCNN uses Depthwise and Depthwise Separate convolution to decrease computation. One of the new Fast-SCNN contributions is the skip connection at the feature fusion step. It helps preserve object boundaries.

There are some problems when using Fast-SCNN with the tomato sucker dataset. Firstly, the resolution of images decreases by 16 times. This leads to the loss of a lot of information because the area of suckers is small compared with the whole image. That is why the result of Fast-SCNN in Section 5 is not good. If we decrease the down-sample rate, the execution time of the “Global Feature Extractor” increases considerably.

## 4. Proposed Semantic Segmentation Neural Network

### 4.1. Proposed Multistage Stage Structure

Inspired by the deep LC method, we proposed our multi-state structure. There are two stages in this structure. We see that the hard regions need a global context for better prediction. For example, if a tomato sucker stands alone, with no stem and no branch beside it, we cannot conclude whether it is a sucker or a small stem. If the deep LC method target is removing easy pixels and adding more feature channels on hard pixels, the main idea of our method is to add more information on hard pixels.

Figure 5 shows the differences between the two structures. In a multi-stage structure, output 1 is not a part of the final output. It detects hard pixels, where stage 1 cannot conclude which class they belong to. Another different point of the new method is the input of stage 2. It includes all information of stage 1 input and hard pixel information that was detected at the previous step. The output of stage 2 is the final output. To save time and detect hard regions efficiently, we use a small convolution neural network at stage 1. On the other hand, a complicated and large convolution neural network would return output with a high score at each pixel, although the prediction may have errors.

### 4.2. Proposed Semantic Segmentation Neural Network

**Figure 7:**
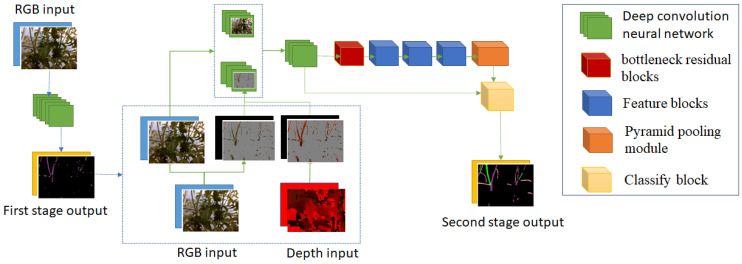
Structure of proposed semantic segmentation neural network.

This paper proposes a high-performance and real-time semantic segmentation neural network. There are three important points in this network. Firstly, we use the multi-structure proposed in Section 3.1. Secondly, we exploit the depth of information to get a better result. Finally, we propose a new convolution neural network structure to build up a semantic segmentation neural network. In recent research [24,25,26,27,28,29], a pair of entire depth images and RGB images are used as the input of the deep convolution neural network with a different moment of fusion of the two data. In our proposed neural network, we present a new depth fusion method. Because of our proposed multi-stage structure, only important regions of depth images are used. The first stage detects the hard regions. The second stage extracts the depth information from these regions and uses it as one of the inputs of this stage. This method eliminates unnecessary depth information which may cause noises in prediction. Figure 7 shows the proposed neural network structure. It includes two stages corresponding with blue and green arrows.

**Figure 8:**
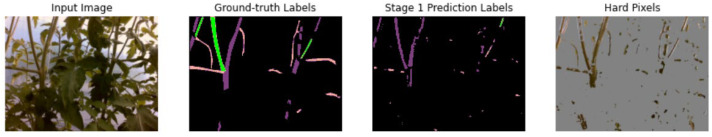
Result of stage 1. Hard Pixels image shows the pixels that stage 1 detected as difficult pixels, which will have more information added in stage 2.

The first stage is a small convolution network. It gets the feature of RGB images quickly and does not make the whole network heavy. The input goes through down-sample convolution layers to reduce the resolution and increase the number of feature channels. This result is up-sampled and followed by a SoftMax activate layer to decode to first stage output. Only easy pixels are detected in this stage, as in Figure 8. The details of the first stage are in Table 1.

Stage 2 gets more information on hard pixels to produce better output. We use the function Premax to get the value of each pixel of output result at stage 1. A pixel with a value greater than t1 is called an easy pixel, and a pixel with a value less than t1 is called a hard pixel. The hard pixels should be getting more information. In our dataset, hard pixels are often the pixels of interest corresponding to the stems, suckers, and branches. In Figure 7, we can see that stage 1 output is not accurate, but it can detect hard pixels. After testing, we saw that t1 = 0.7 gives the best result. The inputs of this stage include normal RGB images, removed easy pixel RGB images, and removed easy pixel Depth images. They are concatenated with the rate 12: 16: 4 and pass through some convolutions before going through a Bottleneck block. Typically, the other network will reduce the resolution and apply more Bottleneck blocks. However, the branches and suckers in the images are thin. The down-sampling and up-sampling operations cause losing their information. Therefore, we propose three different Feature blocks to extract image features at this step. Feature_s1 and Feature_s2 are two convolution block extracts feature with different kernel sizes and different groups. Feature_d extracts feature with different dilation, kernel, and group size. As an alternative for using thousands of feature channels, we use the different combinations of kernel size, group size, and dilation size to get different characteristics of images. Finally, we use the Pyramid Pooling block to get global context, followed by a Classify block. The details of each block and structure of stage 2 are in Table 2, Table 3, Table 4, Table 5, Table 6 and Table 7. The output of this stage is the final output.

We restored the depth distance of pixels and then calculated the average distance. The new values of the distance matrix are calculated as below:

$$D_{ij}=d_{ij}\;-\;\frac{\sum_{i}^{n}\sum_{j}^{m}d_{ij}}{n\;\times \;m}$$

The *d_ij_* is the distance of each pixel, *D_ij_* is the average distance, *n* is the high of an image, and *m* is the width of an image. *D_ij_* value describes the different distances between the objects in an image. This value does not depend on the position of the camera.

Although the proposed neural network has a more complicated structure, the number of parameters of its model is less than Fast-SCNN, which is presented in more detail in Section 5. The lowest resolution is less than eight times the original images, higher than Fast-SCNN. This makes the prediction result more correct.

### 4.3. Proposed Estimation Method for Correct Tomato Sucker Detection

This study’s target is tomato sucker detection, so we developed a method to count how many tomato suckers are detected by semantic segmentation neural network predictions. This method has four main steps:

1. Get two matrices of only sucker labels from the prediction and ground-truth labels. These matrices have values of 0 or 1.
2. OpenCV Connected Component Function is used to separate sucker areas. The sucker regions are noisy regions if they are smaller than a threshold t. After many experiments, we found that t = 160 can remove most noises. Besides, a sucker must be large enough to be removable by a robot.
3. Convert the ground-truth sucker matrix into a list of sucker matrices by OpenCV Connected Component Function.
4. Calculate the sum of each item in the ground-truth label matrix list and the prediction matrix. If the max value of the result equals 2, the prediction is correct.

**Figure 9:**
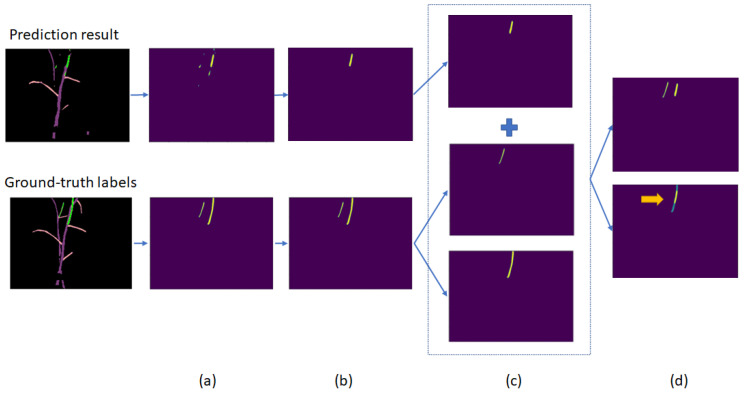
Four steps of detecting the right sucker prediction. (**a**–**d**) is the result of step 1, step 2, step 3, and step 4, respectively. The yellow arrow shows the collapse of the prediction label and ground-truth label. In this case, one true sucker is found.

Figure 9 gives an example of counting correct sucker prediction. Although the prediction result has three different sucker regions, only one is true, or the model detects only one of the two suckers of the input image.

This method is used to evaluate the performance of semantic segmentation neural networks on tomato sucker detection in our experiment. It is an example of how tomato suckers are handled from the output of semantic segmentation neural networks.

## 5. Experiment and Discussion

### 5.1. Setup Experiment

In this part, we demonstrate our model performance on three different tasks. First, we focused on accuracy and speed by comparing our proposed neural network with Fast-CNN and Deeplabv3_resnet50 implemented by the Torchvision library. We chose Fast-CNN because it is a real-time network proven to be better than the other real-time networks [19]. Deeplabv3 is not a real-time network, but it is one of the best segmentation neural networks at the moment. Secondly, we prove that using depth information and multi-stages improves the model’s accuracy by comparing three proposed neural network versions.

Secondly, we focus on detecting suckers, so we used the method proposed in Section 4.3 to calculate the percentage of correctly detected suckers to rank models.

**Figure 10:**
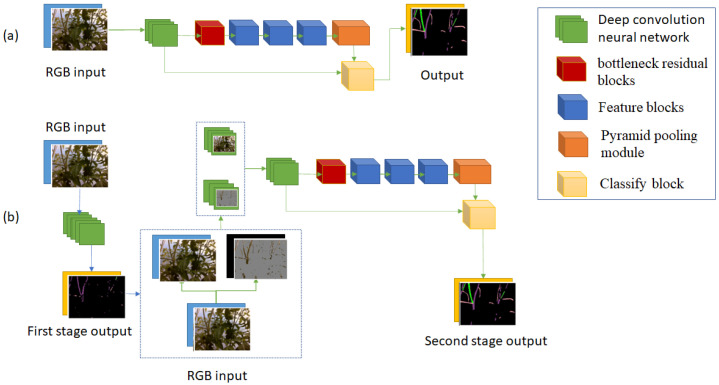
(**a**) is the structure of Version 1 which has one stage, and its input is only RGB images. (**b**) is the structure of Version 2 which has two stages, and their inputs are only RGB images.

Finally, to prove the benefit of using depth information and a two-stage model, we build two more semantic segmentation neural network versions, which are modified from the proposed neural network. Version 1 is the network with the structure of the second stage and no depth information. Version 2 is the network that has two stages but no depth images. The structure of Version 1 and Version 2 are shown in Figure 10.

We write our program in python 3.9 and use PyTorch 1.8 and TorchVision 1.9 library to train and test on a machine with GPU Nvidia Geforce RTX 3090 with CUDA Version 11.2.

We use some augmentation techniques on RGB images: blurring, horizontal flipping, scaling up with a random rate from 1.0 to 2.0, color channel noise, and brightness. Since these operations do not change objects’ spatial structure, the depth information is still correct for new RGB images.

Because our proposed neural network has two stages, the way of training is different. Firstly, we optimized the first stage by using only stage 1 backward to get the best weight of stage 1. Then we optimized only second stage 2 to get the best output. We did not use pre-train weight during training. After many experiments, we found that the network has the best result with batch size = 12, epochs = 40, and learning rate = 0.025, decreasing gradually with each iteration with a rate = 0.997 on the tomato dataset.

We used 80% of images in the dataset for training, 10% for evaluation, and 10% for testing the three models.

### 5.2. Experimental Result 

In this experiment, we use Class IOU (intersection over union), which is very popular to evaluate the accuracy of semantic segmentation neural networks, percentage of tomato sucker detection, and Fps (frames per second) to assess the neural networks.

Table 8 shows the best result of DeepLab V3, Fast -SCNN, and the proposed neural network. The proposed neural network has the smallest number of parameters. It has the best IOU score at 64.05%. Its finding sucker score is lower than DeepLab V3 finding sucker score but much higher than Fast-SCNN finding sucker score. Its Fps is less than Fast-SCNN Fps but is much higher than DeepLab V3 Fps. This result proves that our proposed neural network has the best performance in real-time networks, and the accuracy of our proposed neural network and the other non-real-time network is approximate in tomato sucker detection.

Table 9 shows that the proposed neural network has the best results. Version 1 with one stage is not as good as the two-stage models, but it is the fastest model at 160.7 Fps. Applying a two-stage network structure makes Version 2 and the proposed neural network run slower. The proposed neural network has a higher IOU score and percentage of sucker detection than Version 2, although both neural networks applied the two-stage structure. It is because the input of the proposed neural network used extra depth information, while version 2 used only RGB images.

There is always a trade-off between accuracy and execution time. If a network runs faster, its accuracy is lower. Table 8 shows that the Fps values decrease gradually from Fast-SCNN to the proposed Neural Network to Deeplab V3 while their values of percentage of sucker detection increase, respectively. Similarly, the speed of Version 1 is the highest, as it has the lowest IOU score and percentage of sucker detection.

The more feature channels the neural network has, the more parameters the model has, and the more accurate and slower it would be. For example, Deeplab V3 has 41,999,705 parameters and an IOU score at 63.88, and it runs at 77.6 frames per second, while Fast-SCNN has 1,135,604 parameters and an IOU score at 55.65 and runs at 188.3 frames per second. However, our proposed neural network has a smaller number of parameters but a higher IOU score because its structure is more complicated. It has two stages, two inputs, and two outputs, so it runs slower and gets high accuracy. This can be seen more clearly when we compare Version 1 and Version 2. Their number of parameters is almost equal. Version 2 has two stages. Version 1 has only one stage, so its Fps is higher than other Version 2, but its IOU score and percentage of sucker detection are lower.

These data tables prove that our proposed neural network has the best balance between time execution and accuracy. To increase the accuracy, this neural network uses more complicated and more information of the input instead of increasing the number of parameters.

**Figure 11:**
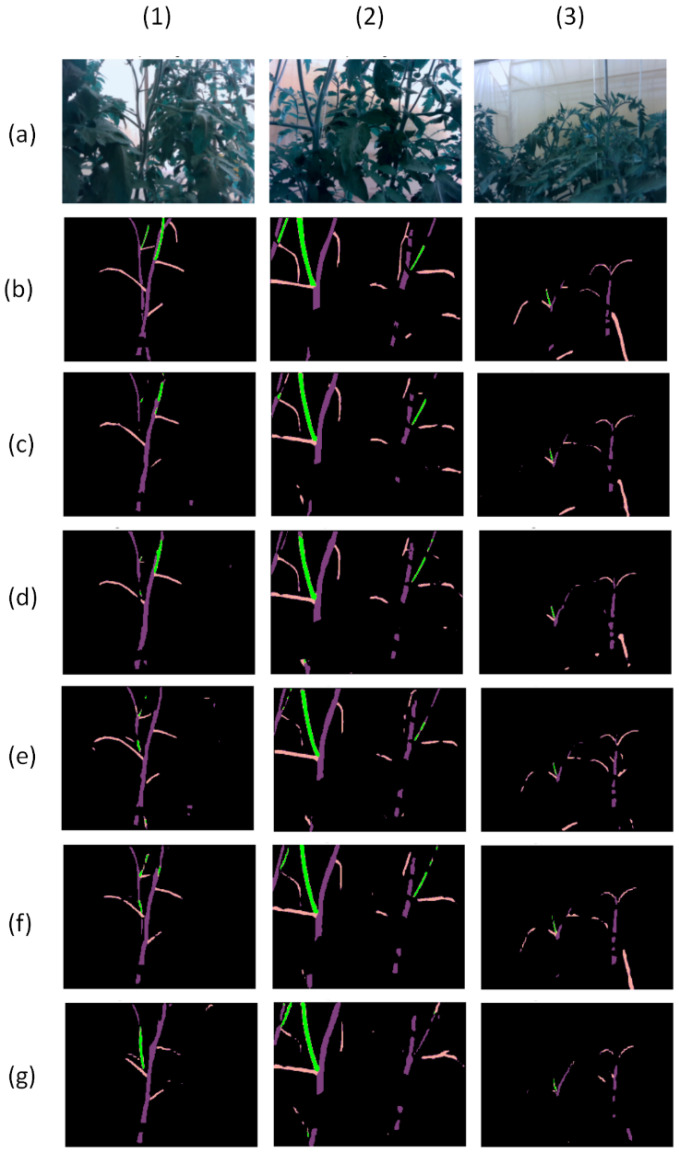
Three examples (1–3) with 6 model results. Row (**a**) is the RGB images, row (**b**) is the ground truth labels, and row (**c**–**g**) are the results of the proposed neural network, Version 2, Version 1, DeepLab V3, Fast-SCNN models.

Figure 11 presents three examples and prediction results of the above six models. The first example is one case where five models misunderstand the behind stem as a sucker, except our proposed in this experiment. In the second example, the proposed neural network and Fast-SCNN can detect two suckers, while Deeplab V3 can detect three suckers. In the third example, three neural networks can detect a sucker, but Fast-SCNN did not recognize lots of branch parts. These examples show that Deeplab V3 can detect the most tomato suckers, the proposed neural network can avoid some errors in some exceptional cases, and Fast-SCNN has the most errors in prediction.

### 5.3. Discussion

The tomato sucker RGB-D images are captured at the greenhouse by the Realsense camera. The depth information could be affected by daylight. The result could be better if we optimize the depth of information. The Dataset should be extended for better training.

If model speed prediction is the most critical factor, we can choose the p neural network Version 1. If the depth of information is not good, Version 2 should be selected.

Although the proposed neural network performs best in our experiment, it can detect 80.2% suckers, and the result usually has noises. To continue developing a method of finding a cut-off point, we must consider some constraints, such as the noise area threshold; the valid suckers are the suckers between stems and branches to eliminate the error.

The proposed neural network has good performance on the tomato dataset and tomato sucker detection but could not have such performance on the other dataset and other functions. Firstly, in our study, there are only four classes to segment. If the number of classes increases, it requires more parameters. Secondly, the proposed neural network does not decrease the resolution of images as much as the other neural networks do during the extracting feature process because stems, branches, and suckers are thin and long. If the target objects are in different shapes, the performance of other networks could be improved.

The training process of the proposed neural network is longer and more complicated than other neural networks. If neural networks have one stage, the training process runs one time to optimize the parameters, while for the neural networks with two stages, the training process must run twice. Firstly, we must set the first stage output as the final output, and the training process could optimize the first stage’s parameters. Then the second output is set as the final output, and the second stage parameters could be optimized by the training process.

## 6. Conclusions

We have proposed an image semantic segmentation neural network to detect tomato suckers in real-time. It has only around 700 thousand parameters and processes images at 138 frames per second. We have demonstrated that our neural network outperforms other real-time neural networks within our scope because of improvement in the network structure. We use a multi-stage structure to make the model have more information on hard pixels. Instead of using many Bottleneck Residual blocks, which lead to memory intensive if the resolution is high, we use three different feature blocks which extract features of images in many different ways. Finally, we also show a new approach to improving the accuracy of the real-time neural network by applying depth information with a multi-stage structure. This research is an important step closer to tomato pruning automatically.

In the future, this study will be extended to develop a method to locate the cut-off point of tomato suckers. We also focus on creating 3D point clouds from tomato plants to perform complicated pruning actions, such as which suckers should be kept or removed.

## Figures and Tables

**Table 1:** Details of State 1.

Operator	Kernel	Stride	Channels
DSConv	3 × 3	2	32
DSConv	3 × 3	2	64
DSConv	3 × 3	1	96
DSConv	3 × 3	1	32
Conv2d	1 × 1	1	4
Upsample (4)	-	-	4
Softmax	-	-	4

**Table 2:** Details of Bottleneck block.

Operation	t	c in	c out	s
Bottleneck	6	64	64	1
Bottleneck	5	64	96	1
Bottleneck	4	96	96	1

**Table 3:** Details of Feature_d block.

Operation	Kernel	Group	Stride	Dilation	Channels
X1: DSConv	3 × 3	256	1	0	256
X2: DSConv	5 × 5	128	1	1	256
X3: DSConv	7 × 7	64	1	2	256
X = add (X1, X2, X3)					256
Conv2D	1 × 1	1	1		96

**Table 4:** Details Feature_s1 block.

Operation	Kernel	Group	Stride	Channels
X1: DSConv	3 × 3	64	1	64
X2: DSConv	5 × 5	32	1	64
X3: DSConv	7 × 7	26	1	64
X = add (X1, X2, X3)				64
Conv2D	1 × 1	1	1	128

**Table 5:** Details of Feature_s2 block.

Operation	Kernel	Group	Stride	Channels
X1: DSConv	3 × 3	128	1	128
X2: DSConv	5 × 5	64	1	128
X3: DSConv	7 × 7	32	1	128
X = add (X1, X2, X3)				128
Conv2D	1 × 1	1	1	256

**Table 6:** Details of Classify block.

Operator	Kernel	Stride	Channels
DSConv	3 × 3	1	64
DSConv	3 × 3	1	32
Upsample (4)	-	-	32
DropOut (0.1)	-	-	32
Conv2d	1 × 1	1	4

**Table 7:** Details of stage 2.

Operator	Kernel	Stride	Channels
I1: Conv2D	3 × 3	1	12
I2: Conv2D	3 × 3	1	16
I3: Conv2D	3 × 3	1	4
I = Concatenate (I1, I2, I3)			
Conv2D	3 × 3	2	32
C1:Conv2D	3 × 3	2	64
Conv2D	3 × 3	2	64
Bottleneck block	-	-	64
Feature_s1	-	-	96
Feature_s2	-	-	128
Feature_d	-	-	256
C2: PPM	-	-	96
Add (C1, C2)			
Classify block	-	-	4

**Table 8:** The comparison of Class IOU, Fps, and Params (the number of parameters) between models on the Tomato dataset.

Models	IOU	Sucker Detection (%)	Fps	Params
DeepLab V3	63.88	82.6	77.6	41,999,705
Fast-SCNN	55.65	57.2	188.3	1,135,604
Proposed neural network	64.05	80.2	138.2	680,760

**Table 9:** Comparison between three different versions of the proposed neural network using depth information and no depth information.

Models	IOU	Sucker Detection (%)	Fps	Params
Version 1	63.41	76.5	160.7	690,596
Version 2	63.74	79.6	138.9	680,832
Proposed neural network	64.05	80.2	138.2	680,760
